# 

**Published:** 1904-01-30

**Authors:** 


### CONTENTS.

**Cancer Research** - - - - - - - - - - - 307

**Voluntary Hospitals and Paying Patients** - - 308

**Annotations** - - - - - - - - - - - - 309

**Notes and News** - - - - - - - - - - - 310

**Hospital Clinics and Medical Progress—**

What We Owe to Experiments on Animals - - - - - 311

On the Etiology of Carcinoma - - - - - - - 313

The Value of Ureteral Catheterisation and Cryoscopy of the Urine - - - - - - - - - - - 313

PROGRESS IN STATE MEDICINE - - - - - - - - 314

PROGRESS OF SURGERY - - - - - - - - - - 315

PROGRESS IN SURGERY OF THE BRAIN AND NERVES - - - 316

**New Appliances and Things Medical** - - - 317

**The Book World of Medicine and Science** - 318

**Hospital Administration—**

St. Bartholomew's Hospital - - - - - - - - 319

Rise and Growth of Vaccination Law—IV. - - - - 323

Glances at the Hospitals - - - - - - - - 324

**Editor's Letter-Box** - - - - - - - - - 325

**The Hospital Library and Charities Bureau** - 326

**The Student of Medicine** - - - - - - - 326

*NURSING SECTION.*

**Notes on News from the Nursing World—**

The Nursing Staff at Osborne House—The New Rule of the Pension Fund—A Poor-law Matron on Stationary Sisters—Church Nurses' Guild at Wimborne House—Edinburgh Royal Infirmary and Outside Probationers—One Trained Nurse for Twelve Sick Wards — Death of a Mission Nurse from Plague — Glasgow Maternity Hospital—Compulsory Attendance at Church—Nurses and Dancing—The Report of the Departmental Committee—Northampton and County Nursing Association—Serious Charges against a Workhouse Matron—The alleged Brawling at Hackney Infirmary — The Nurse and the Curate — District Nurses and Insanitary Dwelling-houses—A Question of Serviettes—Short Items - - - - - - - - - - - 239-242

**The Nursing Outlook** - - - - - - - - - 243

**Lectures upon the Nursing of Mental Diseases** 244

**The Nurses of St. Marylebone Infirmary** - 245

**The Superintendent Nurse at Ladywell Workhouse, and her Duties** - - - - - - - 247

**Infirmary Nurse Burnt to Death** - - - - - 248

**Conference on the Requirements of the Midwives Act in Rural Districts** - - - - - 248

**Appointments** - - - - - - - - - - 249

**Everybody's Opinion** - - - - - - - - 249

**The Nurses' Bookshelf** - - - - - - - - 250

**Where to Go** - - - - - - - - - - - 250

**Echoes from the Outside World** - - - - - 251

**Notes and Queries** - - - - - - - - - 252

**For Reading to the Sick** - - - - - - - 252

